# Qualitative analysis of feasibility of recruitment and retention in a planned randomised controlled trial of a psychosocial cancer intervention within the NHS

**DOI:** 10.1186/s13063-018-2728-y

**Published:** 2018-06-22

**Authors:** M. Duncan, A. Korszun, P. White, G. Eva, on behalf of the SURECAN investigators, Kamaldeep Bhui, Liam Bourke, Trudie Chalder, Jennifer Deane, Sandra Eldridge, John Gribben, Louise Jones, Paul McCrone, Adrienne Morgan, Damien Ridge, Rebecca Roylance, Steph Taylor, Mohamed Thaha

**Affiliations:** 1 Centre for Psychiatry, Wolfson Institute of Preventive Medicine, Queen Mary University of London, London, EC1M 6BQ UK; 2 Department of Clinical Sciences, Brunel University London, Uxbridge, UB8 3PH UK

**Keywords:** Cancer, Psychosocial, Qualitative analysis, Recruitment, Randomised controlled trial, NHS

## Abstract

**Background:**

The randomised control trial (RCT) is the most rigorous method of evaluating interventions. Recruitment is often slower and more challenging than expected. The aim of the current paper is to understand the feasibility of recruitment within the NHS and the barriers and motivators to recruitment from the perspective of patients and healthcare professionals (HCPs).

**Methods:**

NHS HCPs were surveyed to establish their willingness to participate. Twenty HCPs were interviewed to establish barriers and motivators to recruitment. Eleven patients were interviewed to understand their willingness to participate. Interviews were analysed using thematic analysis.

**Results:**

HCP interviews identified key barriers to recruitment: practical barriers included workload and time; clinical barriers included terminology and concern that the trial implied criticism of their current practice; and patient barriers included gender and cultural factors. Motivators to recruitment included: regular communication between research and clinical teams; feedback on findings; and patient and individual benefits for clinicians. Patient interviews suggested that participation in a trial of a psychosocial intervention would strengthen existing coping skills and develop mechanisms for those who were struggling.

**Conclusions:**

Survey results demonstrated that recruitment to an RCT of a psychosocial intervention for people living with and beyond cancer would be feasible within the NHS if specific barriers are addressed. From a clinician point of view, barriers should be addressed to improve recruitment, particularly training and education of clinicians and clear communication. From a patient perspective, interventions and RCT should be tailored to target those not routinely represented in RCTs.

## Background

In randomised control trials (RCTs), participants are randomly assigned to two or more clinical interventions to reduce bias. The RCT is seen as the most scientifically rigorous method of evaluating the effectiveness of interventions and is widely regarded as the gold standard in experimental research design [1]. RCTs are important to establish the evidence base for clinical practice in the NHS [2]. Recruitment targets are set to avoid study findings being statistically underpowered, which may lead to a Type I or Type II error with researchers erroneously detecting an effect where none exists or failing to detect an effect that is, in fact, present. Such errors can result in interventions being provided that lack benefit or, conversely, patients being denied interventions that could significantly improve their health and quality of life (QoL) [3].

Recruitment to trials is frequently much slower and more challenging than expected, with up to 66% of trials not achieving their target sample size [4] and 53% of trials being required to extend the recruitment period, which increases costs and delays the application of research findings to clinical practice [5]. Reasons for unmet targets and delays included issues with local clinical arrangements, with some trials reporting pre-identified centres not participating as planned [5]. Losses of eligible participants were attributable to refusals by patients in 25% of trials and refusals by physicians to recruit in 29% of trials [4]. Achieving set recruitment targets is of paramount importance for the success of a RCT. An understanding of the factors that influence recruitment is therefore vital when planning a RCT.

When considering what may increase motivation to recruit, a monetary incentive is considered less important than factors such as interest in the research, patient benefit and good communication [6]. A systematic review of effective mechanisms to increase the recruitment activity of clinicians involved in RCTs suggested the use of qualitative research to identify and overcome barriers to recruitment, clinicians having a reduction in expected clinical workload when working on RCTs, and training being provided [7]. Good communication with the research team, a clear beneficial effect on patients and clinical practice, an understanding of the purpose of the research, and the individual benefit for clinicians aided recruitment, while HCPs’ perception that barriers are patient-related and out of their control was a barrier [7].

The ProtecT (Prostate testing for cancer and Treatment) RCT used qualitative methods to analyse recruitment discussions and data from interviews with potential participants. Findings suggested that within recruitment to the trial, recruiters had difficulty discussing equipoise and presenting treatments equally, often inadvertently using terminology difficult for patients to interpret [8]. The ProtecT trial successfully optimised recruiters’ communication strategies to increase recruitment rates and demonstrated the benefits of qualitative methods being included either at the feasibility stage of a RCT, or fully integrated into the RCT [9]. The Quartet (Qualitative research to improve recruitment to randomised controlled trials) study collaborated with trial management groups to improve recruitment in trials experiencing poor recruitment by focusing on the way that trials were presented to potential participants [9].

Recruitment to trials with patients with mental health problems face similar issues to trials in general, but there are additional considerations which are specific to this population [10]. The five main reasons for recruitment difficulties in such trials include: misconceptions about trials; a perception of lack of equipoise; HCPs misunderstanding the trial arms and eligibility; and staff paternalism regarding their role as a ‘carer’ [10]. These findings have relevance for planning a RCT of a psychosocial intervention for people living with and beyond cancer.

The aim of this research was therefore to establish the feasibility of recruiting participants to a RCT of a psychosocial intervention designed to improve the QoL of those living with and beyond cancer. In particular, it examined:

1. the willingness of clinics across the NHS to participate in such a RCT;
2. barriers and motivators to recruitment, with a particular focus on cultural barriers;
3. patients’ perceptions of potential recruitment to such a trial.

## Methods

### Survey of HCPs

The approval for the survey as a service evaluation was gained via Bart’s Health NHS Trust (Reg. No. 6131). The four-item scale used was drawn from a larger 22-item standardised survey [11]. The questionnaire items can be found in Table 1. Questions were asked to all participants regardless of their response to item 1. The short survey was sent to a range of professional bodies to try to capture all relevant HCPs involved in the management of cancer patients working in a range of clinical settings. These included the Association of Cancer Physicians, the UK Oncology Nurses Society, the Royal College of Radiologists, the UK Breast Intergroup, British Psychological Society and the Association of Coloproctology of Great Britain and Ireland. The professional bodies distributed the survey to their members via an email link that was open from August to December 2015. The participants completed the survey through the online tool SurveyMonkey (www.surveymonkey.com). Descriptive analysis was used to create proportions with the survey data.

**Table 1:** Survey response to participating in a randomised control trial

	Yes	No	Not sure
1. *Would your unit be interested, in principle, in participating as a centre in a randomised controlled trial of an intervention to improve quality of life of those living with and beyond cancer?*	71% [118]	5% [8]	24% [40]
2. *In principle, would you be willing to release one or two members of nursing or therapy staff, for three days in total, for training in the intervention and delivery of therapy?*	55% [91]	7% [11]	38% [64]
3. *In principle, would you be willing to allow the staff who have been trained to then run the therapy in their unit?*	68% [110]	3% [5]	29% [48]

Participants who responded yes to question 1 were asked how many days a HCP could be spared from their service for training; 73 participants responded, with a mean of 1 day (SD = 1.15)

### Interviews with HCPs

The approval for the interviews with HCPs as a service evaluation was gained via Barts Health NHS Trust (Reg. No. 7070). HCPs who were potential recruiters working within Bart’s Health NHS Trust and University College Hospital were first approached by email to participate in a 10–15-min semi-structured in-person or telephone interview. Consent was sought to record the interview. HCPs were asked about the feasibility of recruiting patients who had finished active treatment; their confidence in (i) explaining a trial, (ii) identifying those with low QoL, (iii) using screening measures; and barriers and motivators to recruiting to a trial within clinics. Interviews were anonymised and transcribed verbatim. Transcripts were analysed using thematic analysis. MD immersed herself in the data, highlighting key sections of text to develop an initial list of codes using the qualitative data analysis software NVivo. The list was then debated with GE to sort into key themes. A table of the full list of themes and codes can be found in the Appendix.

### Interviews with patients

Ethical approval was obtained for this part of the study (Health Research Authority NRES Committee East of England, REC reference: 15/EE/0198 IRAS project ID 168246). Patients who had completed active treatment for cancer were approached in Bart’s Health NHS Trust outpatient clinics and asked whether they would participate in a single semi-structured interview to explore their potential willingness to participate in a trial of a psychosocial intervention aimed at improving QoL. Patients were recruited consecutively but with the aim of including people diagnosed with different types of cancer. To be eligible to participate, patients must have finished active treatment and be aged > 18 years. Patients were excluded if they were not able to speak English. The interviews lasted around 40–90 min and were recorded and transcribed. Analysis proceeded as for the interviews with HCPs.

## Results

### Survey of HCPs

There were a total of 278 responses, of which 166 had completed all four questions resulting in a 60% completion rate. One hundred and eight of the 154 (70%) acute NHS trusts were represented in the survey. We received responses from the Association of Cancer Physicians, the UK Oncology Nurses Society, the Royal College of Radiologists and the UK Breast Intergroup. No response was obtained from The British Psychological Society and the Association of Coloproctology of Great Britain and Ireland. We were unable to calculate an absolute response rate as the respective professional bodies acted as intermediaries in the process in order to protect the privacy of their members and handled dissemination of the survey link. The survey responses to questions asking about participating in a trial can be found in Table 1.

### Interviews with HCPs

In total, 20 HCPs participated in the interview: six Clinical Nurse Specialists and 14 Consultants. HCPs were working in a variety of areas of cancer: nine colorectal, six breast, three head and neck, one haematology and one prostate cancer clinician. Thematic analysis revealed four key themes: practical barriers; clinical barriers; patient barriers; and motivators to improve recruitment.

#### Practical barriers

HCPs identified barriers to recruitment that were out of their control, such as under-resourced staff, limited time within clinic, workload concerns in a busy clinic and the recruitment to other ongoing trials.

*One of the things we are facing at the minute are just how busy clinics are and there’s lots of the new technology coming in like new electronic systems, which means clinic time is squeezed even more. So, what I am certainly finding is I am spending less time with patients and more time at a computer screen, and I am sure that would have a knock-on effect potentially with trials.*

#### Clinical skills barriers

Clinical skills barriers included factors relating to the clinicians perceived ability to promote a RCT. The terminology used and equipoise of clinicians was raised as an issue to recruitment:

*I think the wording would probably need to be changed – I think those words are all very understandable to us as clinicians and scientists but if I said to my average patient, how is your quality of life, they would not necessary know what that means so I think there would need to be some description.*

Other clinical barriers that were highlighted included the risk of clinicians viewing a RCT, especially in psychology, as criticism of their current practices:

*The main barrier was that because we think we have quite a good set up already one of the nurses was a bit sort of suspicious of the study that it was here to sort of investigate what we do.*

The final barrier from clinicians was remembering to mention a RCT to patients within a busy clinic, especially with multiple RCTs to recruit from:

*The difficult bit is remembering and identifying the people who and remember to ask.*

#### Patient barriers

It was a general consensus among clinicians that there would be a certain group of patients that would simply not be interested in a RCT or psychological support:

*There are some patients you will find will want to access everything and there are some patients that will just want to go away and forget it all.*

However, some HCPs identified specific potential barriers relating to the patients included demographic factors such as gender and cultural factors:

*Now I do not want to be sexist but it’s just how I find it, with head and neck cancer it’s very much still such a male-dominated cancer but often they do not like these sorts of things that touch on the psychological aspects, because they are a man and they have got to be strong.*

*It’s not language, it’s culture because people have a lot of people of non-European origin, non-western European origin, intrinsically they do clinical studies, they enjoy it. So, our recruitment is always lower than it should be, just because of that cosmopolitan effect.*

Furthermore, HCPs suggested that RCTs should focus on educational requirements and mental health problems that may prevent a patient from completing extensive outcome measures:

*Quite a lot of our patients have mental health problems, which might mean they do not want to engage in this sort of thing.*

*A significant amount of our patients are from a low socio-economic background and do not always have great educational skills. But in an interview, they would be fine but I know sometimes they struggle with the long questionnaires.*

#### Motivators to improve recruitment

After identifying the barriers to recruitment, HCPs highlighted factors that may improve recruitment. These included regular contact with the research team and/or research staff within clinics:

*We certainly know the best recruiting studies are the ones that have either the research assistant or whoever actually in clinic reminding clinicians of the study, making sure we are asking the questions and giving the patients information.*

The HCPs were more specific about the contact with the research team and felt updates on patient benefits would improve motivation:

*Feedback from patients about how it helped them fed to the recruiting clinicians so we can see how it’s being helpful.*

HCPs felt that communication was also key throughout the whole clinical team surrounding the patients. The clinicians should be aware of the RCT and actively promote it within clinics:

*Making sure all members of the team whether it be me, a CNS, a ward nurse or a radiographer are saying the same thing. Because it’s almost that attrition and you say it enough times to a patient and they sort of give in. So I think that’s an important thing to make sure we are not saying different things and it’s not just one of us continuously saying it but it’s the team.*

Important factors to promote HCPs recruiting patients included both personal benefit such as ‘*being acknowledged in papers*’ and wider patient benefits:

*Just the fact that trials help in the future, so it’s just the fact that every trial is successful and will help many more patients.*

HCPs felt that more in depth preparation for recruitment to a RCT trial may help, such as ‘*more written information, and probably short teaching sessions*’.

### Interviews with patients

Eleven patients completed the interview: four head and neck cancer patients; four haematology patients; and three breast cancer patients. Demographic information can be found in Table 2.

**Table 2:** Patient demographics

Gender	
Female	64% [7]
Male	36% [4]
Ethnicity	
White British	55% [6]
White Other	27% [3]
Asian Indian	9% [1]
Black Caribbean	9% [1]
Occupation status	
Employed	36% [4]
Retired	36% [4]
Missing data	27% [3]
Age (years)	
45–54	25% [3]
44–64	33% [4]
65–74	33% [4]
Missing data	8% [1]
Treatment received	
Surgery	8% [1]
Chemotherapy	17% [2]
Radiotherapy	8% [1]
Transplant	8% [1]
Surgery and radiotherapy	17% [2]
Missing data	42% [5]

Patients’ opinion was divided on whether they would personally participate in the intervention, but they believed it should be provided to those that need the support. Thematic analysis resulted in four main themes for those that would take up the intervention—positives of the intervention, timing of the intervention—and for those that would not—current coping mechanisms and positives of the intervention for others.

The patients that said they would personally be interested in taking part felt that the intervention would improve aspects such as fatigue, returning to work and depression:

*If people are feeling really tired, I think for them that would be good because it’s something positive because I think it might be quite easy to look at the negatives and hang on to them.*

*I wanted to get back to work, but I really did not want to face anybody, because I am on reception so I did not want the constant, ‘How are you feeling? Duh-duh-duh-duh-duh.’ It’s like, yes, I needed to just get back to normal. Yes, it’s the initial first going back, the first week was hard.*

*If they have a couple of follow-up things then it might be you won’t… you will be depressed but you won’t be depressed as intense[ly].*

These patients further suggested that the timing of the intervention being offered was important:

*If it was offered to me when first discharged I may have considered it at the time, but as that wasn’t offered at the time then….*

*The feeling of elation is unbelievable, to be told that you are free of cancer… I wish I had not have had to have that experience but to have had it you cannot describe that feeling and that may override everything else that’s going on up here at that moment in time. So it might be that this needs to settle down a bit, so a month or so later say ‘Look, just, if you feel that you want it, here’s our contact number, get in touch.’ These people might cope with it perfectly well anyway.*

The patients that said that they would not be personally interested tended to report current coping mechanisms such as acceptance and exercise:

*I probably would not but only because of the fact I have someone that I talk to and I think I am working on that generally, accepting that you cannot control everything in life.*

*I think I am fine because I think I have accepted I cannot chew salad with people, cannot do that.*

*I think I am quite lucky that I feel into gym and that is my let up, but I think for other people, yeah, if people are feeling really tired, I think for them that would be good because it’s something positive because I think it might be quite easy to look at the negatives and hang on to them.*

However, they felt that the intervention should be available to those that may need it:

*In my particular case I don’t think so, but in general I think it would be a good idea. But those who don’t have that therapist and counselling would be very good, but those are… because I’m very outward, I do go out and talk to people and make myself busy and everything, but some people are not like that. So for those kinds of people it would be nice for people go and have a chat because instead of being a lonely person they can go and chat with somebody and tell their problems and somebody is there to listen to them and you get advice on that. I think that would be very helpful for patients. Not for me.*

*I think if you, I think it should be offered. Certainly to say, give people the opportunity, explain to them that they might not want it straight away, but say, you know, leave the door open for them and say to them ‘Look, you might not think about it now, you’ve probably got so many other things on your mind’.*

*That’s not me. That is not me. Sometimes group meetings are not me because I want positivity, I do not want negativity.*

## Discussion

The current findings combined survey data from NHS cancer clinics across the UK, HCP interviews and patient interviews regarding the feasibility of recruitment to a RCT of a psychosocial intervention to improve QoL of those living beyond cancer. The survey found that conducting a RCT within the NHS across the UK may be feasible, with most services agreeing to be involved, to allow staff training and the running of an intervention within their service.

The interviews with the HCPs were generally positive, with all participants welcoming RCTs within clinics. The results replicated previous findings of the practical barriers to recruitment including workload concerns, time and under-resourced staff [7]. Similarly, the current findings support previous research demonstrating barriers to recruitment that involved clinical skills such as difficulty discussing equipoise and the use of terminology that is difficult for patients [8]. However, the current findings advanced previous research by suggesting that HCPs were often protective of their current service provision and sometimes saw RCTs as a criticism of their practice. This is an interesting finding as it suggests that to improve recruitment and clinical arrangements an important focus is to acknowledge current practice and explain the purpose of RCTs in providing an evidence base for current practice in specific clinics to provide a case for providing equal care across the UK.

A further important finding was that patient barriers included demographic factors, suggesting that future RCTs should do more to incorporate those that are perhaps underrepresented in cancer QoL research, such as men and those from ethnic minorities. Furthermore, language, educational levels and mental health concerns may be considered a barrier when faced with extensive written outcome measures – which should be addressed in RCT designs to ensure generalisability of findings. Researchers both within and outside of the NHS would benefit from keeping in mind patient barriers when designing their research to improve generalisability of findings and benefit patients that are often underrepresented in research and psychological care.

Participants identified factors that may increase motivation that are simple and easy to incorporate into RCT research, such as regular contact and communication from the research team and throughout the clinical team, regular feedback on the findings and success of the trial, and in line with previous research [7] the importance of the effect of the trial on patients and the individual benefit for clinicians. This finding is high in external validity as all RCTs would benefit from introducing these ideas to increase motivation and recruitment and retention in trials.

The findings from the patient interviews further suggest that a RCT would be welcomed, for those that would benefit – suggesting interventions should be tailored to a patient’s individual needs. The findings demonstrate that those that cope well already use coping mechanisms that would be developed throughout a psychosocial intervention; and those that do not would benefit from support in these key areas of work, exercise and acceptance.

### Limitations

All HCPs who participated in the interviews were working within clinics which were aware of the planned trial and within clinics that provided strong psychological support following active treatment – which is not always the case [11]. Therefore, the current findings based on HCPs already within strong research trusts may overestimate the positive response to recruitment of centres throughout the NHS more generally. Similarly, the patients interviewed were those that volunteered to take part in research and reported a positive experience of care, which may provide a biased sample of patients that perhaps represent those less likely to struggle post treatment and those less likely to be recruited.

### Future research

The SURECAN team have successfully received NIHR funding to develop the intervention further and then conduct a multicentre RCT of a tailored psychological intervention for people who have undergone successful treatment for cancer, but who have a low QoL. The current findings suggested that a RCT was feasible within the NHS and informed the research team how best to improve recruitment and retention within the trial. The team will use the findings of the current study in the new research programme, in particular in acknowledging current practice, providing training and information to HCPs about the purpose of the RCT and will be feeding back findings to the clinical team. The success of these strategies will be revealed in our recruitment rates.

## Conclusions

Survey results demonstrated that recruitment to a RCT of a psychosocial intervention for people living with and beyond cancer would be feasible within the NHS if specific barriers are addressed. From a clinician point of view, barriers should be addressed to improve recruitment, particularly training and education of clinicians and clear communication. From a patient perspective, interventions and RCTs should be tailored to target those not routinely represented in RCTs.

## Appendix

**Table 3:** Initial list of codes and themes from the HCP interviews

Themes	Codes	Example quotes
Practical barriers	Workload concerns Under-resourced staff Time in clinic concerns Set up of clinics Clinics busy Other trials ongoing	I think one of the things we are facing at the minute are just how busy clinics are and there’s lots of the new technology coming in like new electronic systems, which means clinic time is squeezed even more. So what I am certainly finding is I am spending less time with patients and more time at a computer screen, and I am sure that would have a knock-on effect potentially with trials. Radiotherapy is the point which the majority of people end their treatment. Now there are ones that do not get radiotherapy and come through us so there is no one shoe fits everyone. So, if we had a properly integrated service, which we hope to provide at the future, but do not at present it would be more straightforward. But it is not integrated now and because it’s not integrated we have problems. At the moment because of the design of our department, the patients are not focused into one single prostate cancer clinic or kidney cancer clinic, we are trying to get the patients in a focused cancer clinic, so the scenario where you will have these patients coming one after the other through. And we have in terms of the trial portfolio there’s several clinical trials ongoing but I do not think any, I think this would be a very good one to recruit patients into because I do not think there will be any conflict with any other research projects going on. A lot of the patients may already be in a clinical trial so are having QoL measures as part of that. So it’s some of the validated QoL ones that are used, validated for head and neck. Yes, and that might sound harsh but the fact is that we probably have potentially half a dozen studies to recruit to and even with the best will in the world and it will not happen with a lot of additional admin to do. One of the biggest thing is our clinics are really busy and we are really short on physical space. So, there’s a temptation people will kind of exhaust things if they think it will delay. But if it’s a matter of asking the screening and saying they will be contacted later I do not see any reason that we could not get good recruitment. It’s very quick process, only 30 s so I cannot see any problem at all.
Clinical skills barriers	Terminology difficult for patients Criticism of current service Remembering	I think the wording would probably need to be changed – I think those words are all very understandable to us as clinicians and scientists and things involved but if I said to my average patient, how is your QoL, they would not necessary know what that means so I think there would need to be some description of what we mean, like are they enjoying their life, are there things they would like to do that they cannot pursue. So, I think it would have to be broken down, and likewise sort of physical vs the mental so maybe use worked examples, but yeah I think they’d be three very good punch points to use. I think more in lay speak because those are terms that roll off our tongue, but if you actually say it to a patient or carer they will be like ‘What do you mean?’ and the likelihood is there will be assumptions made and therefore we won’t get the right information The main barrier was that because we think we have quite a good set up already, one of the nurses was a bit sort of suspicious of the study that it was here to sort of investigate what we do or if you see what I mean. But X was quite good in that he was here to help us to give us evidence based on what we do and that no patient would receive less than the current standard of care. Again, I think addressing those concerns as most of the work will be done by nursing staff not medical, addressing those concerns and focusing on those would be the best way, reiterating it is a study to help us, to show that what we already do in some alloyed fashion is already effective given us an evidence base to show it’s cost-effective and all those supplementary issues. We are keen to get going and be involved in studies like this That bit is easy, the difficult bit is remembering and identifying the people who and remember to ask actually.
Patient barriers	Patient transport Patients with MHP Group of patients unengaging with psychology Gender barrier Cultural or language barriers Education levels Individual patient response	And a lot of, a sizeable proportion, shall we say 30% come from further afield So they are often an hour away by public transport. Patients who, quite a lot of our patients have mental health problems, which might mean they do not want to engage in this sort of thing. Myself and X felt that there’s definitely going to be a group of patients that will, for whatever reason, not want to engage. The patients themselves, despite you know, you could sell them whatever but they do not want to participate. In terms of patients coming forward, I think there are just some patients an intervention is just not their kind of thing. There are some patients you will find will want to access everything and there are some patients that will just want to go away and forget it all. Another thing that sometimes impacts on these things is especially male patients, now I do not want to be sexist but it’s just how I find it, with head and neck cancer it’s very much still such a male dominated cancer but often they do not like these sorts of things that touch on the psychological aspects, because they are a man and they have got to be strong and these sorts of things. So I think that and that was something I talked to X about that with a lot of these patients that would be suitable for this, we know they need help but they do not and it’s how we actually bridge that gap and try and get them into an important study like this and ultimately help them Yes, rather than just, some of them just immediately shut down. I have sometimes mentioned patients going to our cancer support centre which is an excellent resource, they offer not only counselling, but complementary therapies and psychological input but often patients will not even let you explain what it’s about its all ‘No, no, I do not want any of that’. It is bridging that initial sometimes very negative impressions of that. The majority of the patients I see, we can say 80–90%, speak enough English to understand and be consented for an operation so I do not think language barrier will be a problem. In the few cases where, I think east European and Middle Eastern, people who are not speaking any English, but they are very few, and regarding cultural, difficult to say, maybe there are some cultures that would accept a little bit less, that would be less keen to participate in a trial. The other thing with barriers for our own group would be language. Non-English-speaking people, we do think about how we will introduce the study to them. We might have to give leaflets in Bengali, for instance. Because it’s extraordinarily cosmopolitan and so people come from many different nationalities. I counted over the years about 80 separate nationalities I have seen and it’s extraordinary, you know most people 20 but 80, that’s something quite different. Most countries you can think of I have seen people from, not all. I have not seen anyone from Chad. It’s not language, it’s culture because people have a lot of people of non-European origin, non-western European origin, intrinsically they do clinical studies, they enjoy it. So, our recruitment is always lower than it should be, just because of that cosmopolitan affect. Head and neck, we have a big Bangladeshi population in London and various other ethnic minority groups and again it’s really again it’s, from your point of view, if patients were, English wasn’t their first language would they be excluded or would you have translation facilities? Patients that do not have English as a first language and require interpreters, not all but a significant amount of our patients are from a low socially economic background and do not always have great educational skills. But in an interview, they would be fine but I know sometimes they struggle with the long questionnaires. I think it’s the patients themselves, how enthusiastic they are about things, because they are all individual so it would be how the patient feels about things. But you know anything that will improve patients’ QoL is worth doing, so you know, we’d be totally committed to it.
Motivators to improve recruitment	Regular research contact Peer support in intervention Patient benefits Individual benefits Factors increasing confidence explaining RCT Ease of recruitment Communication throughout team Research member in clinic Reminders Feedback from patient Academic consultants	And I think an academically driven consultant is a great help, which is the case here. Feedback from patients about how it helped them fed to the recruiting clinicians so we can see how it’s being helpful. Positive feedback from our patients saying it had improved them, obviously that would motivate us. If we had notices up to remind us. Also making sure all members of the team, whether it be me, a CNS, a ward nurse or a radiographer, are saying the same thing. Because it’s almost that attrition and you say it enough times to a patient and they sort of give in. so I think that’s an important thing to make sure we are not saying different things and it’s not just one of us continuously saying it but it’s the team that’s looking after the patients that are saying it. Ease of recruitment is the biggest motivation, that’s number 1 We have not got time to do additional things in clinic and this is a simple issue, if it would be a very substantive disincentive if there was a lot of paperwork to do and any paperwork of any sort would be potentially a problem. Make the process as easy as possible and you’ll recruit more patients. The key thing is essentially we generally are happy towards any kind of research as long as it’s not (unheard) making us run around emailing, calling, wait a second wait here, two hours, there will be someone coming or going through paper work or explaining things. That is not what I can do that would be possible with more written information, further information and probably short teaching sessions of what would be expected around the time it will all kick off. I'd want more information and to be kept up to date and things like that but yeah no problems with discussing trials with patients Your name on the paper would be good, would be an incentive. If you say you are recruiting enough patients to the study, you will be part of any scientific production coming out of it, it depends. But obviously that would be an incentive, being acknowledged in the paper is always, generally speaking, an incentive for the contributors. For CNSs in our team the advantages of them being seen to participate in recruitment of trials is obviously a good thing on their CV and it gives them a wider scope for their job as well – all of the advantages that come with being involved in any kind of research. Just the fact that trials help in the future, so it’s just the fact that every trial is successful and will help many more patients in the future. You know it may well be that by identifying these patients early on they may not need so many appointments later on because you deal with a lot of the issues and concerns they may present with later. I think the motivation should be there already, especially within oncologists, I think its interweaved into our beings that the only way to improve the care of our patients and mark the quality of our care is through trials, so I think that’s the one thing. I think lots of people can make lots of excuses but ultimately it’s about quality of care and I think some people may need hand held because it is additional work on an increasingly busy job but I think, my hope is my colleagues see it as I do, that it is as important as giving the radiotherapy or delivering the chemotherapy to patients, and there’s no role in oncology if you are not looking to better things all the time As long as there’s a clear process for referral and that it’s beneficial for the patients. Well sometimes I have found is sometimes getting other patients of a similar background and age and things have a chat with them, but sometimes seeing other patients on the ward in a similar situation. I guess having someone from the research, your end of things keeping regular contact with us would help, these things do drift sometimes if we do not get any feedback and regular contact, that’s always helpful. So, you need to have a dedicated research resource to allow clinicians to interact. And when I took over that trial we were in the bottom of recruitment as no one was taking an interest. I spoke to the CI and now they have got a research nurse that comes to the clinic, sits there. So, I spend the first 15 min of my clinic sitting with them and identifying patients. And then I sit with the patients and introduce it to them, which means they are more receptive to the research nurse. Half of the research nurse job is done. As a consultant, you do have a lot of power, because they trust you more. It will probably help to have someone with a link to the study in clinics. We certainly know the best recruiting studies are the ones that have either the research assistant or who ever actually in clinic reminding clinicians of the study, making sure we are asking the questions and giving the patients information. So that is what helps a CNS or a research person around in a surround that is not clinical outpatient basis so it is like in more informal. Time is the only factor, the only problem. I think rather clinic in breast, it would be useful to have the research fellow there because they have so many cases and it is a bit different. The key point is the MDT and the MDT coordinator, and managing the MDT, so maybe we have quite a lot for research with, for example, UCH on cancer study in preop patients and what they were doing. There was a research fellow at every MDT that was sitting there, coming with us and listening to all the cases, and then he says particularly when the stuff at the end or during the consultation, ‘Well, if you are happy I will do the CAT study for this patient’. We say ‘OK, fine’ and essentially they were arranging something straight away – the patient had the CAT scan within the research at UCH. But obviously they were present constantly and we never object, we never say ‘No, I do not want my patient to go through’, but there was always someone that was there if they were saying to us can you identify every single patient, yeah I am sure that if you occasionally we would not identify the patient as maybe we forget about the study with so many studies going simultaneously we do not think who has all the characteristics to be involved within the study. With my experience, with previous studies, with a research nurse everything goes well and then we do the follow-up 12 months or 24 months later, when we do not have a questionnaire then it all becomes a nightmare. So, I think the most important barrier is to have someone delegated to do all that study, a centralised research person doing all that, then obviously would be easier. So, if I understand you correctly, we would be required to fill out a questionnaire – because what would be ideal is if you had a member of staff present within our clinics. Is that possible?

## Abbreviations

HCPs: Healthcare professionals
IRAS: Integrated research application system
NHS: National Health Service
ProtecT: Prostate testing for cancer and Treatment study
QoL: Quality of life
Quartet: Qualitative research to improve recruitment to randomised controlled trials
REC: Research ethics committee
